# Updates on the Systemic Treatment of Advanced Non-melanoma Skin Cancer

**DOI:** 10.3389/fmed.2019.00160

**Published:** 2019-07-10

**Authors:** Keiji Tanese, Yoshio Nakamura, Ikuko Hirai, Takeru Funakoshi

**Affiliations:** Department of Dermatology, Keio University School of Medicine, Tokyo, Japan

**Keywords:** squamous cell carcinoma, basal cell carcinoma, extramammary Paget's disease, merkel cell carcinoma, adnexal carcinoma

## Abstract

Non-melanoma skin cancers (NMSCs), which represent a diverse group of cutaneous malignancies, are the most common forms of human neoplasia. The incidence of these diseases is increasing due to a number of factors, including that of increasing human lifespans. The majority of NMSCs are basal cell carcinomas (BCC) and cutaneous squamous cell carcinomas (cSCC), with the remainder being various rare skin cancers, including extramammary Paget's disease (EMPD), Merkel cell carcinoma (MCC), and several skin adnexal carcinomas. Of these, MCC usually shows aggressive behavior with a high mortality rate. On the other hand, BCC, cSCC, EMPD, and skin adnexal tumors usually show an indolent clinical course and metastasize only rarely. Nevertheless, the metastatic forms of these tumors commonly lead to poor patient outcome. A definitive management strategy for the treatment of advanced NMSC has not been established, mainly due to their rarity and lack of reliable information based on well-controlled randomized trials. Chemotherapeutic regimens for treatment of these diseases have been mainly based on the observations of isolated, small case series or clinical trials with a limited numbers of patients. However, accumulating evidence regarding their pathobiological backgrounds as well as recent advances in molecular biotechnology have facilitated the development of novel drugs for treatment of these diseases. Over the past decade, the U.S. Food and Drug Administration has approved several molecular targeting therapies, including Hedgehog inhibitors for BCC, monoclonal antibodies targeting anti-programmed death ligand-1 and anti- programmed cell death 1 (PD-1) for MCC, and anti-PD-1 for cSCC. Here, we review their clinical utility and discuss updated systemic treatment strategies for advanced NMSC.

## Introduction

Non-melanoma skin cancers (NMSCs) are the most common forms of human neoplasia, with more than 3 million newly diagnosed cases estimated to occur in the USA every year (1). NMSCs represent a diverse group of skin tumors, including cutaneous squamous cell carcinoma (cSCC), basal cell carcinoma (BCC), extramammary Paget's disease (EMPD), Merkel cell carcinoma (MCC), and skin adnexal carcinomas. Of these, BCC and cSCC account for the majority of NMSCs (75–80% and 20–25% of all NMSC cases, respectively) in Australia (2, 3). In addition, the incidence of these disease is increasing. The incidence rates of BCC and cSCC increased by 145% and 263%, respectively, from 2000 to 2010 in the United States of America (USA) (4). These increases are associated with several factors, including raised awareness of NMSC in the general population, increased number of patients undergoing surgical treatment with confirmed histopathology, improved registration, transition of patient population toward the elderly, and increased exposure to ultraviolent (UV) radiation (5–7). Therefore, NMSC has now become a substantial economic burden (8, 9).

NMSCs show differences in progression and metastatic behavior according to each cancer type. MCCs are highly aggressive malignancies with high mortality rates (10). The 5-year overall survival (OS) rate of MCC patients with localized disease in USA was reported to be 55.6%, with historical 5-year OS rates of 35.4 and 13.5% for patients with nodal and distant metastatic disease, respectively (11). On the other hand, BCC, cSCC, and EMPD generally have a favorable prognosis with surgical resection, especially when detected in the early stages. While its precise clinical behavior is unclear due to its rarity, skin adnexal carcinoma is also considered to have low metastatic potential (12). However, once metastasis occurs, the prognosis of these tumors becomes extremely poor. The median OS period of metastatic BCC was reported to be 10.0 months (range, 0.5–108.0 months) after the detection of metastasis (13). The median progression-free survival (PFS) and OS of stage IV cSCC patients were reported to be 0.67 and 2.19 years, respectively, and the 5-year survival rate was 26% in USA (14). The median OS of EMPD patients with distant metastasis was reported to be 1.5 years with a 5-year survival rate of 7% in Japan (15). While chemotherapeutic agents and treatment strategies for these patients were mainly based on the results of isolated small case series or clinical trials in limited numbers of patients, accumulation of pathobiological evidence as well as advances in molecular biotechnology are facilitating the development of novel drugs and therapeutic regimens. This review presents a summary of updates on the treatment of advanced NMSC based mainly on the results of clinical trials with high evidenced level.

## Basal Cell Carcinoma

**Figure 1:**
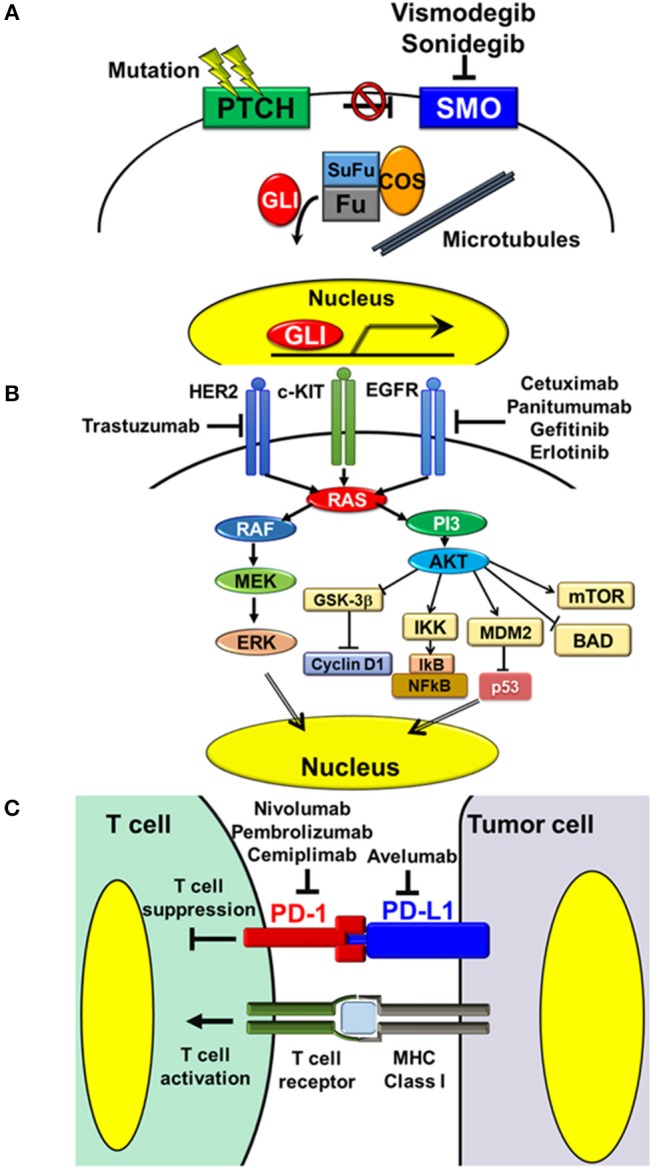
Targeting pathways and molecules in the treatment of NMSC. **(A)** Hedgehog signaling pathway. Activation of Hedgehog signaling is initiated by the cell-surface protein, SMO, which is inhibited by another cell-surface protein, PTCH1. Binding of the Hedgehog ligand to PTCH1 releases this inhibition and thereby activates the pathway. Mutations in *PTCH1* result in loss of its inhibitory function, while mutations in *SMO* lead to constitutive signaling activation. Vismodegib and sonidegib are oral small molecule inhibitors of SMO, which block HH signaling activation. **(B)** Receptor tyrosine kinases and downstream MAPK and PI3-AKT signaling pathways. Aberrant overexpression or mutations of receptor tyrosine kinases, such as EGFR and HER2, cause activation of downstream signaling pathways, thus triggering several tumorigenic processes, including cell proliferation, cell survival, and resistance to apoptosis. The monoclonal antibodies, cetuximab and panitumumab, and the oral small molecules, gefitinib and erlotinib, inhibit the activity of EGFR. The monoclonal antibody, trastuzumab, inhibits the activity of HER2. **(C)** Interaction between T cells and tumor cells via the PD-1/PD-L1 axis. PD-1/PD-L1 interaction inhibits activation of T cell functions, including Th1 cytokine secretion, T cell proliferation, and cytotoxicity. Inhibition of PD-1/PD-L1 interaction with the anti-human PD-L1 antibody, avelumab, and anti-human PD-1 antibodies, nivolumab, pembrolizumab, and cemiplimab, releases these inhibitions and thereby activates the cytotoxic effects of T cells on tumor cells.

BCCs are common skin cancers arising mainly from the basal layer of the epidermis. Clinically, they tend to appear on sun-exposed skin, especially on the face and neck (16). Generally, BCCs are slow growing and have low metastatic potential. However, deeply invasive or large lesions >10 cm^2^ in diameter may show metastasis (17). The estimated metastasis rate ranges from 0.0029 to 0.55%, and common metastatic sites are regional lymph nodes, lungs, bones, skin, and liver (13, 17). Pathobiologically, activation of the Hedgehog (HH) signaling pathway has been shown to play a critical role in the majority of cases and is recognized as a therapeutic target (18–20). It was first characterized by identification of a germ line mutation in the *patched homolog 1* (*PTCH1*) gene in basal cell nevus syndrome, which was then reinforced by the discovery of mutations of *PTCH1, smoothened homolog* (*SMO*), and other genes related to the HH signaling pathway in sporadic BCC (20, 21). In general, activation of HH signaling is initiated by the cell-surface protein, SMO, which is inhibited by another cell-surface protein, PTCH1 (20). Binding of the HH ligand to PTCH1 prevents this inhibition and thus activates signaling. Mutations in *PTCH1* cause loss of its inhibitory role, and mutations in *SMO* release the inhibition resulting in constitutive signaling activation (Figure 1A) (22–24).

## Treatment of Advanced BCC

### Chemotherapeutic Agents

Before the emergence of molecular target therapies, metastatic BCC had been treated with several conventional cytotoxic chemotherapies. However, only a few small case series reported the efficacy of these treatments, due to the rarity of metastatic BCC. In a review of 12 reported cases treated with platinum-containing regimens, five showed complete response (CR) and four showed partial response (PR) (25).

### Molecular Targeting Agents

Two molecular targeting agents are currently available for treatment of advanced BCC, i.e., vismodegib and sonidegib, which were approved by the U.S. Food and Drug Administration (FDA), USA in 2012 and 2015, respectively. Both are oral small molecule inhibitors of SMO, which block HH signaling activation (19) (Figure 1A). One open-label trial of vismodegib showed a response rate (RR) of 68.5% for 1,119 cases of locally advanced BCC (laBCC), including a CR rate of 33.4% and PR rate of 35.1%. The RR in 96 metastatic BCC (mBCC) cases was 36.9%, including 4.8% CR and 32.1% PR. The median PFS was 23.2 months in laBCC and 13.1 months in mBCC (26). In a randomized trial, 12-month administration of sonidegib at 200 mg/day showed RR of 57.6 and 7.7% in 66 laBCCs and 13 mBCCs, respectively. Disease control rates, including cases with stable disease, were 91.9% in laBCC and 92.3% in mBCC (27, 28). In a meta-analysis of 18 reports, the overall response rates (ORR) were similar for vismodegib and sonidegib in laBCC (68.8 vs. 56.6%, respectively), but the complete RRs were markedly different (30.9 vs. 3.0%, respectively). In mBCC, the ORR of vismodegib was 2.7-fold higher than that of sonidegib (39.7 vs. 14.7%, respectively). With regard to side effects, muscle spasms, dysgeusia, and alopecia were noted at similar frequencies for both agents, and their combined prevalence rates were 67.1, 54.1, and 57.7%, respectively (29).

The antifungal drug, itraconazole, has also been reported to inhibit HH signaling activity by acting on SMO (30). In an open-label exploratory phase II trial, 42.1% of cases showed reduction of tumor size and re-epithelization (8/19 cases) (31). Tumor area was reduced by a mean of 24%. Further trials are required to determine its clinical utility.

## Cutaneous Squamous Cell Carcinoma

Cutaneous squamous cell carcinomas (cSCCs) are the second most common skin cancer in NMSCs. In addition to UV irradiation, several risk factors may cause cSCC, including ionizing radiation, chemical carcinogen exposure, chronic wounds or scars, immunosuppression, infection with certain genotypes of human papillomavirus (HPV), and genetic abnormalities of genes involved in DNA repair (32–34). While the majority of these lesions can be treated by surgical resection, a small proportion will develop metastasis with significant morbidity and mortality (35). Of all metastatic cases, around 80% are localized in the regional lymph nodes, with the remainder showing distant metastasis in multiple organs, including the lungs, liver, brain, bones, and skin (32).

Pathobiologically, cSCCs are characterized as tumors with high mutational burdens. Exome-level sequencing of eight primary cSCCs revealed approximately 1,300 somatic single-nucleotide variations per cSCC exome (1/30,000 bp) (36). Among them, several genomic alterations were reported to show strong associations with the development and progression of cSCC. One study regarding single nucleotide and copy number variations in DNA samples extracted from metastatic cSCCs indicated that TP53 (79% of cases), NOTCH-1/2/4 (69%), and CDKN2A (48%) genes were frequently altered and several signaling pathways, including the RAS/RAF/MEK/ERK1/2 MAPK pathway (MAPK pathway) and phosphatidylinositol 3/AKT pathway (PI3-AKT pathway), are frequently activated (45% of cases) (37). Inactivating mutation of TP53 due to UV irradiation has been reported to be a cause of cutaneous malignancy with loss of programmed cell death in abnormal squamous cells (38). Loss of NOTCH-1 was reported to be associated with disease progression (39). CDKN2A encodes two cell cycle regulatory proteins, p16 and p14, loss of which causes aberrant mitosis (40). In addition, several reports indicated aberrant overexpression or mutations of epidermal growth factor receptor (EGFR) in cSCC samples. EGFR belongs to the receptor tyrosine kinase (RTK) family that activates several downstream signaling pathways, including MAPK and PI3-AKT pathways, and is involved in various cellular processes, including cell proliferation and survival (41, 42) (Figure 1B).

## Treatment of Advanced cSCC

### Chemotherapeutic Agents

Chemotherapeutic agents commonly utilized in treatment of advanced cSCC include bleomycin, platinum, anthracycline, taxanes, interferon alpha, 5-fluorouracil, and its precursor drug capecitabine (43–49). While there have been a number of reports of the efficacies of chemotherapeutic regimens combining these agents, the level of evidence was generally poor as the trials had several limitations, such as small sample size, heterogeneous patient populations, and lack of randomization.

### Molecular Targeting Agents

Based on the frequent aberrant overexpression or mutations of EGFR in cSCC, the utility of agents targeting EGFR has been suggested (Figure 1B). The efficacy and safety of the monoclonal antibodies, cetuximab, and panitumumab, and oral small molecules, gefitinib and erlotinib, have been reported in several clinical trials and retrospective studies in small patient populations. Cetuximab was evaluated prospectively in a phase II study in 36 cases of advanced cSCC, including three metastatic and 33 locally advanced cSCCs in France. The ORR was 27.8%, including two cases of CR and eight cases of PR, the rate of stable disease was 41.7% (15/36 cases), and the overall disease control rate was 69.4% (25/36 cases). The most common grade 3 and 4 toxicities were infection (22.2%) and bleeding from the tumor (11.1%) (50). In a retrospective study in 34 locally advanced cSCC patients in France treated with cetuximab alone or in combination with platinum and 5-fluorouracil as neoadjuvant therapy, the RR of cetuximab alone patients was 55.5% (5/9 cases), including three cases of complete histological response, while that of combination therapy patients was 92% (23/25 cases), including 15 cases of complete histological response (51). The utility of panitumumab was evaluated in a small phase II study in 16 patients with advanced cSCC in Australia. The ORR was 31.3% (5/16 cases), including two cases of CR (52). While monoclonal antibodies showed certain RRs, oral small molecules showed limited efficacy for advanced cSCC. In phase II studies conducted in USA, gefitinib and erlotinib only showed PR rates of 15% (5/40 cases) and 10.3% (3/29 cases), respectively (53, 54).

### Immune Checkpoint Inhibitors

Recent advances in cancer immunotherapy have provided new therapeutic approaches involving blocking of immune checkpoints. In particular, antibodies targeting PD-1 and its ligand, programmed cell death ligand 1 (PD-L1), have been reported to have prospective efficacy in various cancer types (55) (Figure 1C). The same applies to advanced cSCC; administration of the anti-PD-1 monoclonal antibody, cemiplimab, showed impressive efficacy. Utility of cemiplimab in a phase I expansion cohort study including 16 metastatic and 10 locally advanced cSCCs showed ORR of 50% (13/26 cases) and a durable disease control rate of 65.4% (17/26 cases). Duration of response exceeded 6 months in 53.8% (7/13 cases) of patients who showed a response. In a phase II study in 59 cases of metastatic cSCC, the ORR was 47.5% (28/59 cases), including four cases of CR, the durable disease control rate was 61.0% (36/59 cases), and the duration of response exceeded 6 months in 57.1% (16/28 cases) of patients who showed a response. By integrating the results of these two studies, the RR of cemiplimab for metastatic cSCC was 46.7% (35/75 cases). As high mutational and neoantigenic burdens have been reported to be strong predictors of responsiveness to immunotherapy, cSCC is expected to be responsive to immune checkpoint inhibitors. Safety assessment evaluation indicated that treatment was well-tolerated. The most common adverse events in the above phase II study were diarrhea (27.1%), fatigue (23.7%), nausea (16.9%), constipation (15.3%), and rash (15.3%). The observed grade 3 and 4 toxicities were infection (22.2%) and tumor bleeding (11.1%). The most common grade 3 and 4 toxicity was pneumonitis (3.4%), and no severe toxicities were present in more than 5% of patients (56). Based on these observations, cemiplimab was approved by the FDA, USA in 2018 for treatment of advanced cSCC.

## Extramammary Paget's Disease

EMPD is a rare intraepithelial adenocarcinoma that principally affects the genital and axillary regions. EMPD typically grows slowly and is diagnosed as an *in situ* lesion, and it generally shows favorable prognosis with surgical resection. However, once it invades the dermis, EMPD easily causes metastasis and the prognosis becomes poor (57). Although its pathogenesis remains to be clarified, it has been reported to resemble breast cancer in its immunohistochemical and molecular profiles (58).

Pathobiologically, human epidermal growth factor receptor 2 (HER2) has been reported be overexpressed in 15–60% of EMPD cases, mainly based on amplification of the *HER2* gene (59–61). HER2 is a member of the RTKs, which regulate several downstream pathways, including MAPK and PI3-AKT pathways (42, 62) (Figure 1B). *HER2* gene amplification causes HER2 protein overexpression, resulting in ligand-independent homodimerization and aberrant activation of downstream signaling pathways (63). Of note, immunohistochemical staining for phosphorylated ERK and phosphorylated AKT, which are signatures reflecting activation of MAPK and PI3-AKT pathways, respectively, are positive even in HER2-negative cases (59, 61). A recent report indicated that 81.3% (13/16 cases) of Japanese metastatic EMPDs are positive for phosphorylated ERK and phosphorylated AKT regardless of the HER2 expression status, and 68.8% (11/16 cases) were positive for both signatures (61). Furthermore, DNA sequence analysis of EMPD revealed that 19% of cases had mutant *RAS* or *RAF* genes and 35% of cases had mutations in *PIK3CA*, which encodes the catalytic subunit of PI3K, or in *AKT1* that activates these pathways, suggesting that these two signaling pathways play critical roles in the pathogenesis of EMPD (64).

Signaling through androgen hormone receptor (AR) was also reported to be associated with the development of EMPD. The AR-positive rate in EMPD has been reported to be 54–90%, and its level of expression was significantly higher in invasive EMPD than non-invasive EMPD (65, 66). Moreover, the expression levels of the androgen-producing enzymes, 5α-reductase and 17β-hydroxysteroid dehydrogenase type 5, were shown to be higher in invasive EMPD than non-invasive EMPD (66, 67). These results suggested an association between androgen–AR signaling and the progression of EMPD.

## Treatment of Metastatic EMPD

### Chemotherapeutic Agents

At present, no chemotherapeutic agents have yet been approved for the treatment of metastatic EMPD. Several cytotoxic chemotherapeutic regimens have been used to treat metastatic EMPD, such as combination of low-dose 5-fluorouracil and cisplatin (FP therapy), combination of 5-fluorouracil, epirubicin, carboplatin, vincristine, and mitomycin C (FECOM therapy), combination of cisplatin, epirubicin and paclitaxel (PET therapy), combination of docetaxel and S-1, docetaxel monotherapy, and S-1 monotherapy (68–76). In general, certain patient populations initially respond well to these therapies. However, the efficacies are usually temporary, and tumors soon recur due to acquisition of resistance. Low-dose FP and FECOM regimens showed RR of 59 and 57% with median PFS of 5.2 and 6.5 months as first-line treatment in Japanese metastatic EMPD patients, respectively. However, the median OS rate was <1 year (68, 69). The DTX monotherapy for Japanese metastatic EMPD patients was reported to show an RR of 58%, but its median PFS and OS were 7.1 and 16.6 months, respectively (70). Nevertheless, modification of the treatment schedule or improvement of the chemotherapeutic regimen may enhance the efficacy. In PET therapy, adjustment of the dosing interval enabled Japanese metastatic EMPD patients to continue treatment by reducing its severe toxicity, while maintaining its high efficacy (76).

### Molecular Targeting Agents

HER2 is recognized as a prospective therapeutic target in cases of advanced HER2-positive EMPD based on the pathobiological similarities to breast cancer and its frequent overexpression in EMPD (Figure 1B). The HER2-specific humanized monoclonal antibody, trastuzumab, is an established treatment option for metastatic HER2-positive breast cancers (77). Several case reports have indicated the effectiveness of trastuzumab in combination with cytotoxic chemotherapeutic agents in EMPD (78–81). Based on these findings, a phase II study of trastuzumab with docetaxel for HER2-positive unresectable or metastatic EMPD (jRCTs031180073) is currently underway in Japan.

For therapy targeting androgen–AR signaling, one case report showed the utility of combined androgen blockade (CAB) therapy consisting of bicalutamide (anti-androgen drug) and leuprolide acetate (LH–RH agonist), which is used in the treatment of prostate cancer. While its efficacy lasted for only 6 months, it significantly reduced multiple bone metastases of EMPD (82).

## Merkel Cell Carcinoma

Merkel cell carcinoma (MCC) is a rare but highly aggressive cutaneous neuroendocrine carcinoma (10). While they are named after sensory Merkel cells in the skin based on their ultrastructural and immunophenotypic resemblance, the true origin of MCC tumor cells remains unknown (83). Risk factors for MCC include age ≥65 years, immunosuppression, previous sun exposure, and Merkel cell polyomavirus (MCPyV) infection (84). The majority of MCCs are caused by the integration of MCPyV into the genome, and the remainder are associated with exposure to UV irradiation (84, 85).

MCPyV is a non-enveloped double-stranded DNA virus that belongs to the polyomavirus family and is highly prevalent in the general population. Primary MCPyV infection usually occurs during childhood and can be detected in the skin of nearly all healthy individuals. Despite its widespread infection, MCC develops in only a small percentage of the population. The viral oncogenes, LT and sT antigens, have been suggested to play important roles in MCPyV-induced tumorigenesis. LT antigen has been shown to bind to retinoblastoma (RB) protein, inactivating its tumor suppressive function (86). sT is the major viral oncogene that contributes to virally induced cellular transformation (87). It can maintain the hyperphosphorylated and inactivated state of the eukaryotic translational initiation factor 4E-binding protein 1 (4E-BP1), ultimately leading to acceleration of cell proliferation and malignant transformation (87). sT also inhibits the cellular ubiquitin ligase, SCFFbw7, and suppresses proteasomal degradation of MCPyV LT and other cell cycle regulators, thus contributing to viral replication and host cell transformation (88). The viral DNA was shown to be clonally integrated into the genome of MCC cells (84).

Integration of MCPyV has also been reported to be associated with immunogenicity of MCC (84, 89). Several reports indicated that MCPyV-positive tumor cells express viral antigens at high levels, which are recognized by the innate and adaptive immune systems (90). In particular, patients with higher immune responses to MCPyV show better disease outcomes than those with modest responses (91, 92). MCPyV-negative MCCs may also have immunogenicity, based on their high mutational burden and neoantigens generated by exposure to UV irradiation. In fact, MCPyV-negative MCCs have more gene mutations than cells in most other cancer types (85). Furthermore, about 50% of MCCs have been reported to express PD-1 on tumor-infiltrating lymphocytes and PD-L1 on tumor cells or infiltrating macrophages (93).

## Treatment of Advanced MCC

### Chemotherapeutic Agents

Several cytotoxic chemotherapies have been preferred as treatment options for advanced MCC. Chemotherapeutic regimens were designed based on those used in small cell lung cancer (94). These include combination of platinum and etoposide, combination of cyclophosphamide, doxorubicin (or epirubicin) and vincristine, and monotherapies of topotecan and etoposide (95). Although MCC shows a relatively high RR to first-line chemotherapy, the response is rarely durable, and resistance develops quickly. One retrospective study has reported RRs of 69 and 57% to first-line chemotherapy for locally advanced and metastatic MCC, respectively, but survival was limited to averages of 24 and 9 months, respectively (96).

### Immune Checkpoint Inhibitors

Based on its high immunogenicity, the application of immune checkpoint inhibitors for MCC has been examined in several clinical trials with promising results. In particular, blockade of the PD-1/PD-L1 interaction prolonged the responses and improved OS of advanced MCC. Avelumab is a monoclonal anti-human PD-L1 antibody, which activates antibody-dependent cell-mediated cytotoxicity as well as blocking PD-1/PD-L1 interactions (Figure 1C). In an international multicenter phase II trial in 88 cytotoxic chemotherapy-refractory metastatic MCC patients, avelumab treatment (10 mg/kg every 2 weeks) showed ORR of 33% (29/88 cases) over the minimum follow-up period of 2 years (median 29.2 months), including a CR rate of 11% (10/88 cases) (97, 98). With regard to safety concerns, only 5% (4/88 cases) of patients had grade 3 adverse events and there were no grade 4 or 5 treatment-related adverse events. In an international, multicenter, single-arm, open-label clinical trial in 39 treatment-naive patients, avelumab showed ORR of 62.1% (29/39 cases) at the minimum follow-up period of 3 months, including CR of 13.8% (4/39 cases) and PR of 48.3% (14/39 cases) (99). Pembrolizumab is a monoclonal anti-human PD-1 antibody, which also blocks PD-1/PD-L1 interactions (Figure 1C). In a phase II study in 26 systemic therapy-naive stage 3 or 4 MCC patients, administration of pembrolizumab (2 mg/kg every 3 weeks) showed ORR of 56% (14/25 cases) including CR in four cases and PR in 10 cases. With regard to safety concerns, grade 3 or 4 adverse events occurred in only 15% (4/26) of patients and these were manageable (93). Based on these reports, avelumab and pembrolizumab have now been approved by the FDA, USA for treatment of metastatic MCC. With regard to other agents targeting PD-1/PD-L1 interaction, the anti-human PD-1 antibody, nivolumab, showed efficacy for MCC. In a phase I/II study in 15 treatment-naive and 10 previously treated metastatic MCC patients, administration of nivolumab (240 mg every 2 weeks) showed ORR of 68% (15/22 cases) including CR and PR in three and 12 of 22 evaluable cases, respectively. PFS and OS rates at 3 months were 82 and 92%, respectively (100).

## Skin Adnexal Carcinoma

Skin adnexal carcinomas are a group of malignancies exhibiting histopathological features of follicular, sebaceous, apocrine, or eccrine differentiation. Pilomatrix carcinoma, trichilemmal carcinoma, and trichoblastic carcinoma are categorized as those showing follicular differentiation, while sebaceous carcinoma is categorized as those showing sebaceous differentiation. Malignant tumors showing sweat gland differentiation include porocarcinoma, spiradenocarcinoma, hidradenocarcinoma, apocrine adenocarcinoma, microcystic adnexal carcinoma, adenoid cystic carcinoma, malignant mixed tumor, malignant cylindroma, digital papillary carcinoma, syringoid eccrine carcinoma, and mucinous carcinoma of the skin (101). The precise molecular pathogenesis of skin adnexal carcinomas is still under investigation. Whole-exome sequencing of sebaceous carcinomas indicated that they can be divided into three clinically distinct classes: pauci-mutational type harboring fewer mutations, UV damage type with a high mutational burden due to UV damage, and microsatellite instability (MSI) type with microsatellite repeat sequence replication errors. Ocular sebaceous carcinoma belongs to the pauci-mutational type, sebaceous carcinomas associated with Muir-Torre syndrome, a hereditary cancer syndrome associated with germline mutations in mismatch repair pathway components, belongs to the MSI type, and UV damage type skin adnexal carcinomas tend to show poorly differentiated histological features (102). HRAS and EGFR have been suggested to play pathobiological roles in some cases of porocarcinoma (103). Expression of EGFR has also been reported in several sweat gland carcinomas (104). In addition, expression of HER2 (105–107) and c-KIT (108, 109) and the activation of MAPK or PI3-AKT signaling pathways have been described in single case reports of several skin adnexal carcinoma types (108–110).

## Treatment of Skin Adnexal Carcinoma

At present, there are neither approved chemotherapeutic agents nor uniform guidelines for the treatment of skin adnexal carcinoma. Therefore, information regarding the utility of systemic treatments for these diseases is available only in published case reports. In general, skin adnexal carcinomas are considered to be relatively chemoresistant, and the prognosis of their metastatic forms is considered to be poor. Although further studies are needed to clarify the usefulness of proposed treatment options, several reports indicated the efficacy of various chemotherapeutic agents used in combination therapy.

### Chemotherapeutic Agents

With regard to metastatic skin adnexal carcinomas with sweat gland differentiation, one report indicated CR for 16 months in one case with a combination of doxorubicin, mitomycin, vincristine, and 5-fluorouracil followed by maintenance combination therapy consisting of cyclophosphamide, vincristine, and 5-fluorouracil (111). Other reports have also shown the efficacy of various other combinations of drugs, including 5-fluorouracil, thiotepa, and cyclophosphamide (112, 113), anthracycline, cyclophosphamide, vincristine, and bleomycin (114), interferon-alpha and weekly paclitaxel (115), and doxorubicin, mitomycin C, vincristine, and cisplatin (116). Notably, the combination of carboplatin and epirubicin maintained CR for 4 years in a case of porocarcinoma (49).

### Molecular Targeting Agents

With regard to targeted therapy, administration of sunitinib, an oral small molecule, multi-targeted RTK inhibitor, stabilized disease progression over 8 months in a patient with metastatic clear cell hidradenocarcinoma and achieved PR for 10 months in a patient with metastatic trichoblastic carcinoma (117). Administration of trastuzumab showed CR for 7 months in a patient with HER2-positive apocrine carcinoma. Moreover, combination of capecitabine and lapatinib, an oral anti-HER2 targeted therapy, in the same patient achieved CR for 6 months after the metastatic lesion developed resistance to trastuzumab (118).

### Immune Checkpoint Inhibitors

The usefulness of targeting PD-1/PD-L1 interaction has also been reported. The results of immunohistochemical analysis indicated that PD-L1 was expressed in 50% (12/24) of ocular sebaceous carcinoma cases (119). Administration of anti-human PD-1 antibodies showed efficacy in patients with metastatic sebaceous carcinoma (120, 121).

## Conclusion

While a standard management strategy for treatment of advanced NMSC has not been established, advances in our molecular biological understanding of NMSC and improvement of drug discovery techniques over the past several decades have facilitated the establishment of novel treatment strategies. Nevertheless, emerging molecular targeting therapies are not necessarily effective for all NMSC patients. Development of further treatment options for NMSC is required, especially for rare forms of NMSC, such as skin adnexal carcinomas.

## Author Contributions

TF wrote the part of squamous cell carcinoma. IH wrote the part of extramammary Paget's disease. YN wrote the part of basal cell carcinoma. KT wrote the part of Merkel cell carcinoma and adnexal carcinomas. KT also unified the format of entire manuscript.

### Conflict of Interest Statement

The authors declare that the research was conducted in the absence of any commercial or financial relationships that could be construed as a potential conflict of interest.
